# Long-term changes of serum chemokine levels in vaccinated military personnel

**DOI:** 10.1186/1471-2172-7-21

**Published:** 2006-09-11

**Authors:** Beda Brichacek, Christophe Vanpouille, Alexander J Trachtenberg, Tatiana Pushkarsky, Larisa Dubrovsky, Gregory Martin, Gary Simon, Michael Bukrinsky

**Affiliations:** 1 Department of Microbiology, Immunology and Tropical Medicine, George Washington University Medical Center, Washington, DC, USA; 2 US Naval Medical Research Center Detachment, Lima, Peru; 3 Division of Infectious Diseases, Department of Medicine, George Washington University Medical Center, Washington, DC, USA

## Abstract

**Background:**

Members of the United States Armed Forces receive a series of vaccinations during their course of service. To investigate the influence of multiple vaccinations on innate immunity, we measured concentrations of a panel of immunomodulatory and pro-inflammatory cytokines in serum samples from a group of such individuals.

**Results:**

Significantly increased levels of macrophage inflammatory protein 1α (MIP-1α), MIP-1β and interleukin 8 (IL-8) were detected. Since these cytokines are known to have anti-human immunodeficiency virus (HIV) activity, we tested the effect of serum from these individuals on HIV-1 infectivity and susceptibility of their peripheral blood mononuclear cells (PBMCs) to HIV-1 infection in vitro. Sera from vaccinated military personnel inhibited, and their PBMCs were partially resistant to, infection by HIV-1 strains tropic to CCR5 (R5), but not to CXCR4 (X4), chemokine receptor.

**Conclusion:**

These findings demonstrate that increased anti-HIV chemokines can be detected in vaccine recipients up to 68 weeks following immunization.

## Background

Viruses and other pathogens express a variety of proteins interfering with the host immune responses to counteract immune surveillance and increase their virulence [1]. The ability of a pathogen to modulate host response to infection and the reaction of host cells to such immunomodulation form an environment that can influence concurrent or subsequent infections by other pathogens [2-5]. A significant role in this immunomodulation is played by the cytokine-chemokine network [6]. For example, infection with mouse lymphocytic choriomeningitis virus abolishes replication of the hepatitis B virus, and this process is mediated by tumor necrosis factor-α and interferon-γ [7]. Co-infection of human lymphoid tissue *ex vivo *with human herpesvirus 6 and CCR5-utilizing HIV-1 results in suppression of HIV infection, a process dependent on herpesvirus 6-mediated upregulation of CC chemokine regulated upon activation in normal T cells expressed and secreted (RANTES), a natural ligand for CCR5 [8]. Upregulation of chemokines was also implicated as a primary mechanism of HIV-1 suppression caused by at least two other pathogens – measles [9] and GB virus C (GBV-C) [4]. Similar to co-infections, immunizations, especially with live vaccines, can change the immune environment and cytokine profile and may have unexpected effects on subsequent infection with an unrelated pathogen.

## Results and discussion

**Figure 1:**
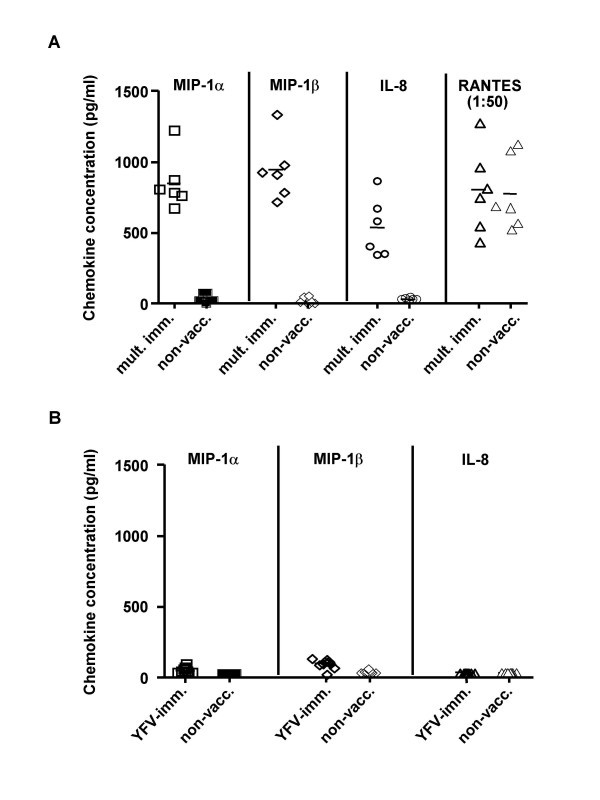
**Serum chemokine levels in study subjects**. A. Chemokine levels in serum samples of multiply immunized military personnel (mult. imm.) and control unvaccinated subjects (non-vacc.) measured by ELISA. Chemokine concentrations are shown for each subject of 2 groups (6 vaccinated and 6 matching unvaccinated individuals). p < 0.001 for MIP-1α and MIP-1β, and p < 0.002 for IL-8. B. Chemokine levels were measured by ELISA in serum samples of subjects vaccinated against Yellow Fever (YFV-imm.) and control individuals (non-vacc.). Results are presented for each subject (10 YFV-immunized and 16 matching controls).

To test whether immunizations have a long-term effect on cytokine profile, we measured, using Luminex technology, concentrations of a number of immunomodulatory cytokines in serum samples from six members of the US military. These individuals received multiple immunizations including vaccines against smallpox (live vaccinia virus), anthrax, typhoid, rabies, and influenza during the 15 month period preceding the blood donation (Table 1). Ten non-military subjects immunized with another live vaccine, against the yellow fever virus (YF), and 16 non-vaccinated individuals of matching sex and age served as controls. The time between the last vaccination and blood donation for military personnel ranged between 14 and 62 weeks (Table 1). Blood from subjects immunized with the YF vaccine was collected 16–24 weeks after vaccination, to allow development of anti-YF immune response [10] and to match experimental group. Importantly, subjects in this group did not receive any other vaccine within a period of 60 weeks prior to YF vaccination. Significantly elevated (compared to unvaccinated group) levels of CCL3 (MIP-1α), CCL4 (MIP-1β) and CXCL8 (IL-8) were found in serum samples from vaccinated military personnel, but not in recipients of vaccine against yellow fever. These results were then verified using ELISA (Fig. 1 and Table 1). No other factor tested, including CCL5 (RANTES, Fig. 1A), CXCL12 (stroma cell-derived factor 1 [SDF-1]), IL-1α, IL-1β, IL-2, IL-4, IL-6, IL-12, IL-15, IL-16, CXCL10 (IP-10), CXCL9 (monokine induced by IFN-γ [MIG]), granulocyte-macrophage colony-stimulating factor (GM-CSF), interferon gamma (IFN-γ), and tumor necrosis factor α (TNFα) varied significantly between multiply vaccinated individuals and unvaccinated subjects.

**Table 1:** Immunization timetable of enrolled military personnel for the 15 month preceding blood collection.

**Vaccination (weeks prior to blood collection)**						**Serum chemokines (pg/ml)**			
**donor**	**smallpox**	**anthrax**	**rabies**	**typhoid**	**influenza**	**MIP-1α**	**MIP-1β**	**IL-8**	**RANTES**

1	57	45	14	14	14	671	981	346	40,866
2	44	nd	nd	nd	nd	874	722	861	63,986
3	60	*	*	*	*	1,219	1,338	669	27,619
4	58	49	ni	49	70, 18	801	791	577	37,571
5	62	ni	ni	ni	ni	760	929	404	48,545
6	62	57, 55, 53	62	55	ni	780	915	348	22,070

*Donor #3 was immunized once against smallpox, once against anthrax, once against typhoid, three times against rabies and once against influenza within 60 weeks prior to blood donation (exact dates of immunizations are not available)

nd – no data available; ni – not immunized.

**Figure 2:**
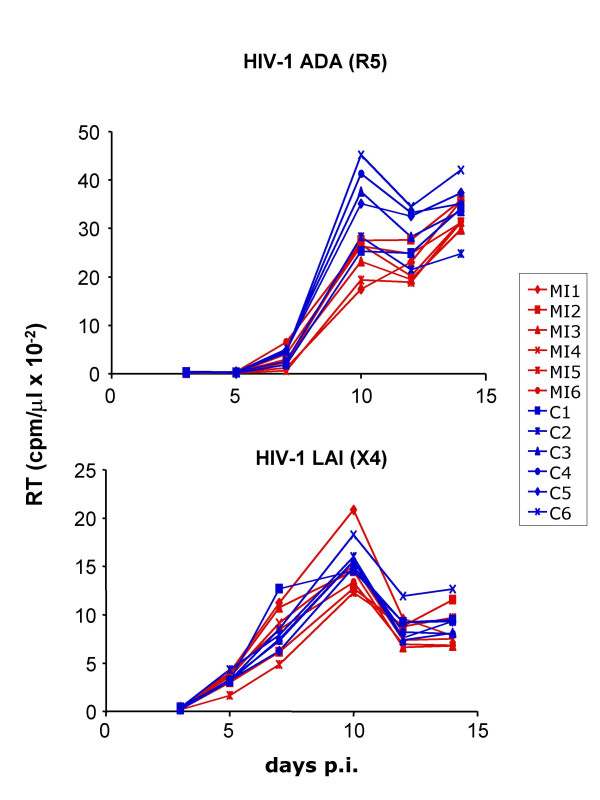
**Suppression of HIV-1 R5 replication by sera from multiply vaccinated subjects**. Replication of R5 (ADA) and X4 (LAI) HIV-1 strains was measured in unstimulated PBMC cultures from unvaccinated donors in the presence of 5× diluted serum from multiply immunized (MI) or control (C) subjects (6 per group). Results are shown for one representative experiment out of four performed with cells from different donors. Higher concentration of the serum or effect on other viruses could not be tested in this assay because of the shortage of the serum available.

MIP-1α and MIP-1β are ligands for the CCR5 receptor [11,12], while IL-8 has been shown to induce CCR5 internalization [13]. Since CCR5 is a co-receptor for the majority of transmitted HIV-1 strains [14,15], the observed cytokine profile could provide resistance to HIV-1 infection through interference with virus interaction with CCR5. To test this supposition, we analyzed the effect of sera from vaccinated and control subjects on replication of R5 and X4 HIV-1 in PBMCs from unvaccinated control donors. PBMCs from 4 different donors were cultured without external activation for 3 days to allow physiological monocyte-dependent activation of T cells and then infected with HIV-1_ADA _(R5 strain) or HIV-1_LAI _(X4 strain) adjusted according to reverse transcriptase activity. Serum from vaccinated military personnel, but not unvaccinated control subjects, provided partial protection of PBMCs against infection with R5, but not X4, HIV-1 (Fig. 2). This result is consistent with previously reported anti-R5 activity of MIP-1α and MIP-1β at concentrations found in the sera of vaccinated subjects [16]. Nevertheless, the possibility remains that some additional soluble factors could also contribute to the observed protection. Interestingly, conflicting reports have been published regarding the effect of CC chemokines on replication of X4 HIV-1 strains, with one group reporting enhancement of X4 HIV-1 replication [17], whereas the other not confirming this effect [18]. We did not observe an enhancement of replication of HIV-1_LAI _by sera from multiply vaccinated subjects, consistent with the report by Cocchi and colleagues [18].

**Figure 3:**
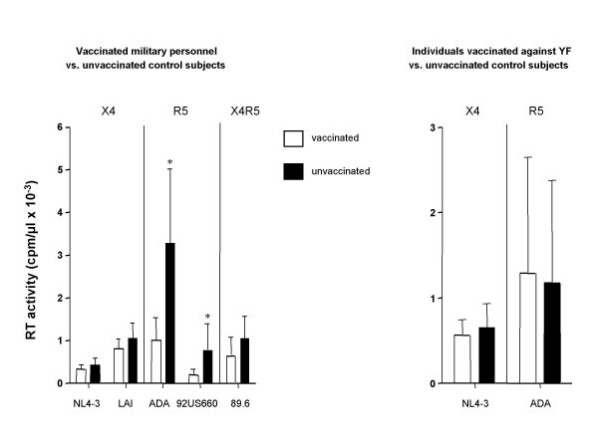
**HIV-1 replication in PBMC cultures from vaccinated and unvaccinated donors**. Graphs show the cumulative viral production during the 2-week cultivation (mean RT activity ± SD) for each group (6 vaccinated military subjects and 6 matching unvaccinated individuals in the left panel; 10 subjects vaccinated against YF and 16 matching unvaccinated individuals in the right panel). Student's *t *test was used to establish statistical significance between groups; *p < 0.05.

We next tested susceptibility of PBMCs from vaccinated military personnel to HIV-1 infection *in vitro*. Similar to the previous experiment, cells were cultured for 3 days without any exogenous activating factor, and then were infected with R5 (92US660 and ADA), X4 (NL4-3 and LAI), or dual-tropic (X4R5) (89.6) strain of HIV-1. Results presented in Figure 3 demonstrate that replication of the R5 viruses was significantly suppressed in PBMC cultures derived from military personnel compared to cultures from unvaccinated control subjects. This difference became even more pronounced when autologous serum was present during the *in vitro *infection (data not shown), consistent with the presence in these serum samples of chemokines with CCR5-specific anti-HIV activity (Figs. 1 and 2). No significant differences between cells from vaccinated and unvaccinated subjects were observed in infections with X4 or X4R5 strains of HIV-1 (Fig. 3). Also, neither R5 nor X4 strains were suppressed in cells from YFV-immunized subjects (Fig. 3). The protective effect against R5 HIV-1 strains was lost when the infected cultures were activated with phytohemagglutinin (results not shown), suggesting that strong activation may overcome the protective effect of immunization. Surprisingly, analysis of CCR5 expression on CD4 positive cells from multiply vaccinated and control subjects did not reveal significant differences (results not shown). This result, however, does not rule out the possibility that the functional activity of CCR5 as an HIV receptor may be impaired. Further studies will be required to investigate the mechanisms of this interesting observation.

Vaccine against smallpox was the only vaccine received by all the military personnel involved in this study within past 15 months, whereas all other immunizations varied between the subjects. That and the fact that subject 5 did not get any other immunization but vaccinia (see Table 1), yet his serum showed the same chemokine profile as serum from other subjects of this group (Fig. 1), suggests that vaccinia virus may be responsible for the observed effect. However, the effect of another immunization(s) received in the more distant past cannot be excluded at the present time. The observed protection is unlikely to be a non-specific effect of immunization with a live vaccine, since vaccination against yellow fever did not alter cytokine profile or affect sensitivity of recipient's PBMCs to either R5 or X4 HIV-1 (right panel in Fig. 3). The uniformly very low levels of MIP-1α, MIP-1β and IL-8 present in the serum of control subjects, which had broad racial diversity, suggests that racial bias (controls were only age and gender matched) is unlikely to be responsible for the observed differences.

## Conclusion

This study provides evidence that some strong immunogens, or their combination, can cause long-term change in serum chemokines and induce resistance of PBMCs to HIV-1 infection. Despite precedence from veterinary medicine [19], the observed long-term increase in cytokine levels induced by vaccination has, to our knowledge, never been reported in humans. Further studies are necessary to determine which vaccine (or vaccine combination) is responsible for the protective effect against HIV-1, but vaccinia is our favorite. Our recent study on PBMCs from another group of military personnel who received vaccinia immunization demonstrated a similar restriction of replication of R5 HIV-1 strains, including a slight enhancement of suppression of R5 HIV-1 strains in the presence of autologous serum of multiply immunized individuals (results not shown). The donors involved in our study were not tested for the presence of GBV-C. However, it appears unlikely that all multiply immunized subjects and none of the subjects in the control groups are GBV-C infected, arguing against the role of GBV-C infection in the observed phenomenon. Nevertheless, such test should be included in any of the future studies devoted to this question. These results warrant a larger study of the effects of multiple immunizations including vaccinia. Knowledge of the mechanisms responsible for such stimulation of innate immunity by conventional vaccines may be used to increase the efficiency of vaccines against HIV-1 and, if found to involve long-term activation of other aspects of innate immunity, could have an impact on immunization approaches.

## Methods

### Human subjects

Multiply immunized study volunteers were recruited from among the US Navy medical personnel. All six individuals were white males, between 35 and 45 years of age. They received multiple immunizations in conjunction with their occupational health program. Six non-military individuals matched by age (within 3 years) and gender served as non-vaccinated controls. The ten subjects vaccinated against yellow fever and the corresponding non-immunized controls were recruited from the GWU Medical Center Travel Clinic and GWU faculty. A non-immunized individual was defined for the purpose of the study as a person who did not receive any vaccination, did not have any major infection, nor was on immunotherapy within past 6 months. Yellow fever vaccinees and control groups were of varied racial background. All individuals involved in the study traveled quite extensively. Studies in human subjects were performed in accordance with the Helsinki Declaration and were approved by the George Washington University Medical Center Institutional Review Board (ref. IRB #100537). All subjects provided written consent for drawing blood.

### Cells and viruses

PBMCs (2 × 10^6 ^cells/culture) were cultured without adding exogenous activating agents for 3 days, at which point the culture medium was replaced with diluted serum from vaccinated or control individuals. After 3 hrs, viral inoculum was added and incubation continued overnight. Infection was performed with R5 (92US660 and ADA), X4 (NL4-3 and LAI), or dual-tropic (X4R5) (89.6) strains of HIV-1. The virus inoculum was adjusted according to reverse transcriptase activity to 1.4 × 10^5 ^cpm per 10^6 ^cells. After an overnight incubation, non-attached virus was washed off in a series of washes with RPMI-1640 supplemented with 10% FBS, 2 mM L-glutamine and 10 μg/ml of gentamicin. In the experiments where exogenous activation was employed, cells were activated with phytohemagglutinin (5 mg/ml for 48 hrs) following the wash step after the infection and cultured in the presence of IL-2 (20 U/ml).

### Chemokine/cytokine analysis

The levels of granulocyte-macrophage colony-stimulating factor (GM-CSF), interferon gamma (IFN-γ), interleukin (IL)-1α, IL-1β, IL-2, IL-4, IL-6, CXCL8 (IL-8), IL-12, IL-15, IL-16, CXCL10 (interferon-inducible protein [IP]-10), CXCL9 (monokine induced by IFN-γ [MIG]), CCL3 (macrophage inflammatory protein [MIP]-1α), CCL4 (MIP-1β), CCL5 (RANTES), CXCL12 (SDF-1), and tumor necrosis factor (TNF)-α in culture medium were evaluated by using the multiplex bead-array assays performed on a Luminex-100 platform (Bio-Plex system – Bio-Rad) as described previously [9]. Briefly, individual Luminex bead sets were coupled to cytokine-specific capture antibodies according to the manufacturer's instructions. The assays were run by using 1,200 beads/set/well in a total volume of 50 μl. For each bead set, 61 beads were collected. Chemokine profiles of MIP-1α, MIP-1β, IL-8 and RANTES were confirmed by specific ELISAs (R&D Systems, Minneapolis, Minnesota) according to the manufacturer's protocol.

### Flow-cytometric analysis

After an overnight incubation in culture media without exogenous activation (see *Cells and viruses*), aliquots of PBMCs were washed three times and stained with monoclonal antibodies (Pharmingen) either as a single staining or in the following combinations: CD4-fluorescein isothiocyanate (FITC)/CCR5-phycoerythrin (PE), CD8-FITC/CCR5-PE and CD4-FITC/CXCR4-PE. Staining with corresponding isotype antibodies was used as a control. Flow-cytometric analysis was performed on BD FACSCalibur. Obtained data were evaluated using FlowJo (Tree Star, Inc) software.

### Statistical analysis

Student's two-tails paired *t *test was used to establish statistical significance between groups.

## Authors' contributions

BB participated in the design of the study, carried out *in vitro *infections, ELISA assays, performed the statistical analysis and participated in writing the manuscript. CV carried out the Luminex assays. AJT assisted with cell fractionation and culturing. TP participated in interpretation of results. LD amplified HIV-1 stocks and performed RT assays. GM recruited military subjects and collected samples. GS recruited YF vaccinated subjects and collected samples. MB participated in the design of the study and drafted the manuscript. All authors read and approved the final manuscript.

## References

[B1] Vossen MT, Westerhout EM, Soderberg-Naucler C, Wiertz EJ (2002). Viral immune evasion: a masterpiece of evolution. Immunogenetics.

[B2] Yoshida A, Maruyama H, Kumagai T, Amano T, Kobayashi F, Zhang M, Himeno K, Ohta N (2000). Schistosoma mansoni infection cancels the susceptibility to Plasmodium chabaudi through induction of type 1 immune responses in A/J mice. Int Immunol.

[B3] Jung S, Knauer O, Donhauser N, Eichenmuller M, Helm M, Fleckenstein B, Reil H (2005). Inhibition of HIV strains by GB virus C in cell culture can be mediated by CD4 and CD8 T-lymphocyte derived soluble factors. AIDS.

[B4] Xiang J, George SL, Wunschmann S, Chang Q, Klinzman D, Stapleton JT (2004). Inhibition of HIV-1 replication by GB virus C infection through increases in RANTES, MIP-1alpha, MIP-1beta, and SDF-1. Lancet.

[B5] Moss WJ, Ryon JJ, Monze M, Cutts F, Quinn TC, Griffin DE (2002). Suppression of human immunodeficiency virus replication during acute measles. J Infect Dis.

[B6] Margolis L (2003). Cytokines – strategic weapons in germ warfare?. Nat Biotechnol.

[B7] Guidotti LG, Borrow P, Hobbs MV, Matzke B, Gresser I, Oldstone MB, Chisari FV (1996). Viral cross talk: intracellular inactivation of the hepatitis B virus during an unrelated viral infection of the liver. Proc Natl Acad Sci USA.

[B8] Grivel JC, Ito Y, Faga G, Santoro F, Shaheen F, Malnati MS, Fitzgerald W, Lusso P, Margolis L (2001). Suppression of CCR5- but not CXCR4-tropic HIV-1 in lymphoid tissue by human herpesvirus 6. Nat Med.

[B9] Grivel JC, Garcia M, Moss WJ, Margolis LB (2005). Inhibition of HIV-1 replication in human lymphoid tissues ex vivo by measles virus. J Infect Dis.

[B10] Lefeuvre A, Marianneau P, Deubel V (2004). Current Assessment of Yellow Fever and Yellow Fever Vaccine. Curr Infect Dis Rep.

[B11] Raport CJ, Gosling J, Schweickart VL, Gray PW, Charo IF (1996). Molecular cloning and functional characterization of a novel human CC chemokine receptor (CCR5) for RANTES, MIP-1beta, and MIP-1alpha. J Biol Chem.

[B12] Combadiere C, Ahuja SK, Tiffany HL, Murphy PM (1996). Cloning and functional expression of CC CKR5, a human monocyte CC chemokine receptor selective for MIP-1(alpha), MIP-1(beta), and RANTES. J Leukoc Biol.

[B13] Richardson RM, Tokunaga K, Marjoram R, Sata T, Snyderman R (2003). Interleukin-8-mediated heterologous receptor internalization provides resistance to HIV-1 infectivity. Role of signal strength and receptor desensitization. J Biol Chem.

[B14] Alkhatib G, Combadiere C, Broder CC, Feng Y, Kennedy PE, Murphy PM, Berger EA (1996). CC CKR5: a RANTES, MIP-1alpha, MIP-1beta receptor as a fusion cofactor for macrophage-tropic HIV-1. Science.

[B15] Blanpain C, Libert F, Vassart G, Parmentier M (2002). CCR5 and HIV infection. Receptors Channels.

[B16] Brandt SM, Mariani R, Holland AU, Hope TJ, Landau NR (2002). Association of chemokine-mediated block to HIV entry with coreceptor internalization. J Biol Chem.

[B17] Kinter A, Catanzaro A, Monaco J, Ruiz M, Justement J, Moir S, Arthos J, Oliva A, Ehler L, Mizell S, Jackson R, Ostrowski M, Hoxie J, Offord R, Fauci AS (1998). CC-chemokines enhance the replication of T-tropic strains of HIV-1 in CD4(+) T cells: role of signal transduction. Proc Natl Acad Sci USA.

[B18] Cocchi F, DeVico AL, Yarchoan R, Redfield R, Cleghorn F, Blattner WA, Garzino-Demo A, Colombini-Hatch S, Margolis D, Gallo RC (2000). Higher macrophage inflammatory protein (MIP)-1alpha and MIP-1beta levels from CD8+ T cells are associated with asymptomatic HIV-1 infection. Proc Natl Acad Sci USA.

[B19] Cox SJ, Aggarwal N, Statham RJ, Barnett PV (2003). Longevity of antibody and cytokine responses following vaccination with high potency emergency FMD vaccines. Vaccine.

